# The complete chloroplast genome of *Calystegia pubescens* ‘Anestia’ Hara 1957 (Convolvulaceae), an endemic species in Asia

**DOI:** 10.1080/23802359.2025.2602225

**Published:** 2026-01-12

**Authors:** Hua Chen, Ji-Si Zhang

**Affiliations:** Liaoning Key Laboratory of Development and Utilization for Natural Products Active Molecules, Anshan Normal University, Anshan, PR China

**Keywords:** *Calystegia pubescens* ‘Anestia’, chloroplast genome, Convolvulaceae, phylogeny

## Abstract

In this study, we conducted a comprehensive sequencing and characterization of the chloroplast genome of the Asian endemic species *Calystegia pubescens* ‘Anestia’. The complete genome is 152,079 bp long, with a GC content of 37.37%. It consists of a large single-copy region (87,891 bp), a small single-copy region (19,888 bp), and a pair of inverted repeats regions (22,149 bp). A total of 126 genes were identified, including 81 protein-coding genes, 37 tRNA genes, and eight rRNA genes. Phylogenetic analysis revealed a close relationship between *C. pubescens* ‘Anestia’ and *C. hederacea*. These results provide valuable genetic insights for species identification and phylogenetic studies within the genus *Calystegia*.

## Introduction

**Figure 1.**
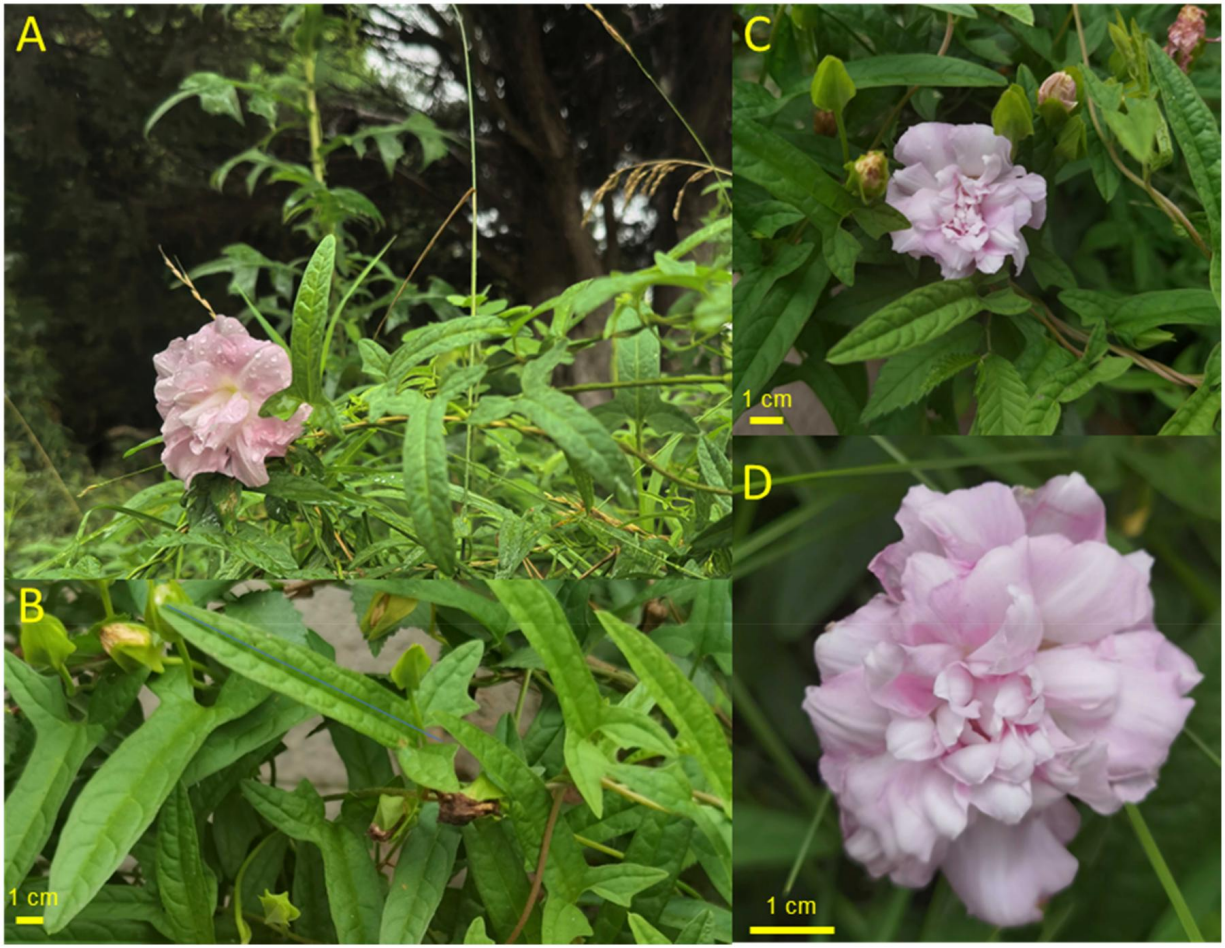
Pictures of *C. pubescens* ‘Anestia’. The key features include stems usually climbing; leaf blade narrowly triangular, parallel sided at middle, strongly lobed at base; corolla pink; Stamens and anthers absent. (A) Habitat (campus of Anshan Normal University, Liaoning Province). (B) Leaves. (C) Stems with flower and flower buds. (D) Flower. All photos were taken by Ji-Si Zhang on July 2025. The species was identified by Ji-Si Zhang.

The taxonomic history of *Calystegia pubescens* ‘Anestia’ has undergone several revisions. Initially, it was described as *Calystegia dahurica* (Herb.) Choisy f. *anestia* (Fernald) Hara (Hara 1957). Subsequently, Fang and Brummit (1995) assigned the name *C. pubescens* Lindl. 1846, which was identified as *C. japonica* Choisy (1854) in Japan. Hara (1957) further distinguished *C. pubescens* from *C. japonica*, and categorized the former as a form of *C. dahurica* (Herb.) Choisy (1845), although the validity of the name *C. dahurica* remains uncertain (Fang and Brummitt 1995). Despite variations in stem hairiness, leaf-base shape, and flower morphology between Chinese *C. pubescens* Lindl and Japanese *C. japonica*, these differences seem to fall within the spectrum of a single species based on a comprehensive analysis of Chinese specimens (Hara 1957) (Figure 1). Yonekura (2005) treated wild *C. japonica* with pink and white flowers as a form of *C. pubescens*. Consequently, *Calystegia dahurica* (Herb.) Choisy f. *anestia* (Fernald) Hara was recognized as a heterotypic synonym of *C. pubescens*, subsequently named *Calystegia pubescens* ‘Anestia’. This form is commonly found in the East Asian temperature region, thriving in diverse habitats such as waste areas, grassy or shrubby slopes, and occasionally as a weed in cultivation (Hara 1957). Notably, the chloroplast genome of *C. pubescens* ‘Anestia’ has not been documented. To address this research gap, we present the complete chloroplast genome of *C. pubescens* ‘Anestia’, offering a valuable genomic resource to clarify the taxonomic position of this species and elucidate the phylogeny of the genus *Calystegia* within the Convolvulaceae family.

## Materials and methods

Leaves of *C. pubescens* ‘Anestia’ were collected at the campus of Anshan Normal University, located in Liaoning, PR China (122.995366°N, 41.076753°E) (Figure 1). A voucher specimen was archived at https://www.gxib.cn/ under the supervision of Zhufang Bin (Contact: 1162004502@qq.com, voucher number IBK00472640). All activities, including sample collection, photography, species identification, and specimen preparation, were carried out by Ji-Si Zhang (Contact: zhangjisi@asnc.edu.cn). This species is not protected, and no special permissions were required.

DNA was extracted from gel-dried leaves utilizing the modified CTAB method (Doyle and Doyle 1987). Subsequent library preparation and sequencing were performed at Personalbio (Shanghai, China) employing an Illumina Hiseq2000 sequencer (San Diego, CA), and approximately 4 Gb of 150 bp paired-end raw reads were obtained. Evaluation of raw read quality was conducted using FastQC v0.11.9 (Brown et al. 2017), followed by filtering out adapters and low-quality reads with Trimmomatic v0.39 (Bolger et al. 2014). Assembly of the clean reads was performed using default parameters in GetOrganelle v1.7.3.2 (Jin et al. 2020). Visualization of scaffolds and their connectivity was accomplished by Bandage v0.7.1 (Wick et al. 2015). The chloroplast genome was annotated and manually verified in Geneious v9.05 (Kearse et al. 2012) with reference to *C. hederacea* (NC_085534). CPGView (http://47.96.249.172:16085/cpgview/view) was utilized for improved annotation and identification of cis- and trans-splicing genes (Liu et al. 2023). Sequence alignments were executed with MAFFT v7 (Katoh and Standley 2013), and Gblock v0.91b was used to eliminate ambiguous regions (Talavera and Castresana 2007). Phylogenetic analyses of the complete chloroplast genomes were performed through Bayesian inference (BI) employing MrBayes v3.2.6 under the GTR + G model (Ronquist and Huelsenbeck 2003), as well as maximum parsimony (MP) methods using PAUP v4b10 with heuristic searches and 1000 bootstrap replicates for support values (Swofford 2003).

## Results

**Figure 2.**
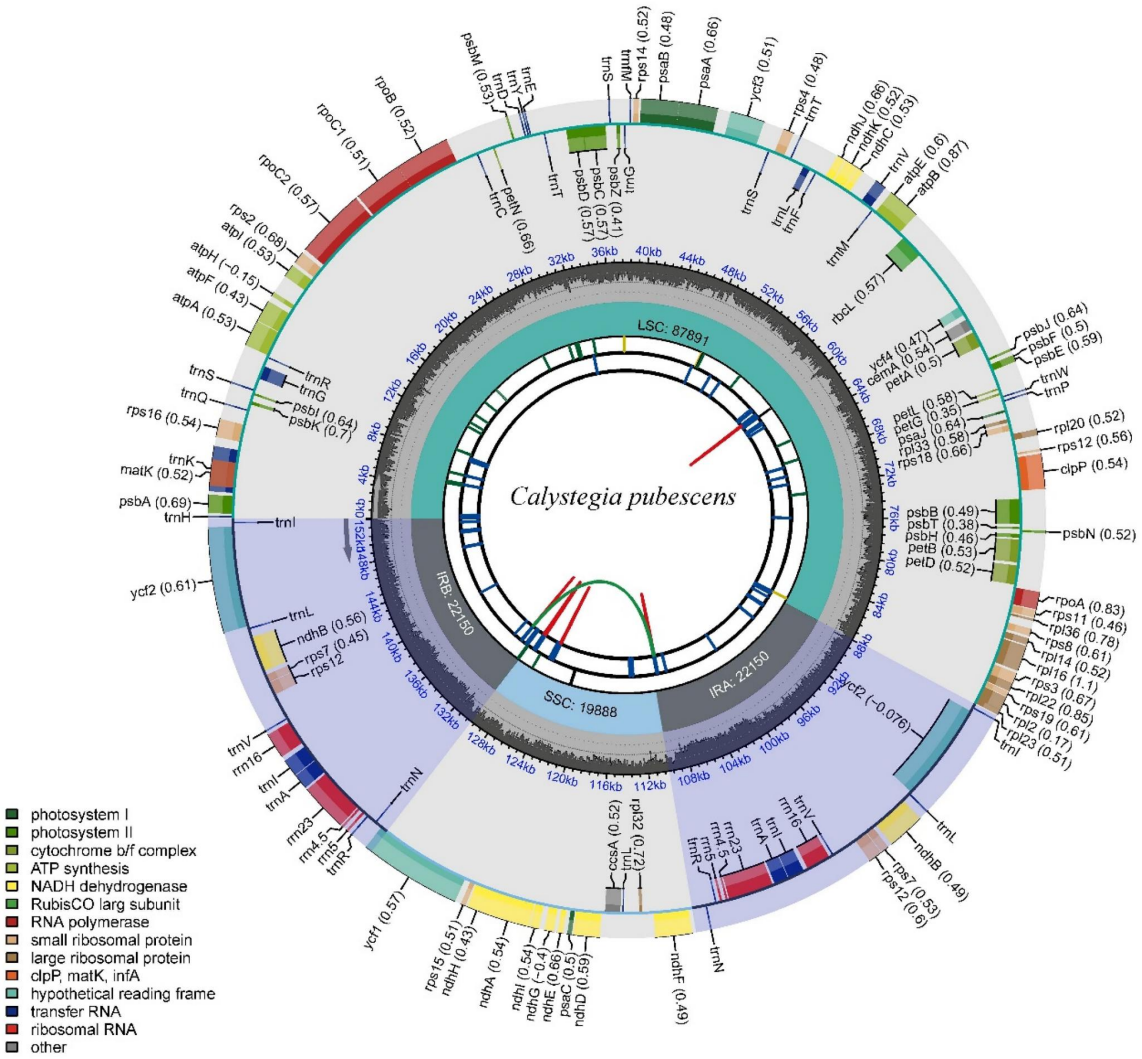
Chloroplast genome map of *C. pubescens* ‘Anestia’. Different function groups of genes are signed according to the colored boxes. LSC: large single-copy; SSC: small single-copy; IRA and IRB: inverted repeat regions. From the inside: the first circle shows the dispersed repeats, the second circle shows the long tandem repeats. The third circle shows the short tandem repeats or microsatellite sequences. The fourth circle shows the genome length of LSC, SSC, and IRs, respectively. The fifth circle shows the GC content along the genome. The sixth circle shows the genes, and the numbers in parenthesis are optional codon usage bias.

The chloroplast genome of *C. pubescens* ‘Anestia’ (GenBank accession number PV938954) was examined for coverage depth, as illustrated in Figure S1. This genome, spans 152,079 bp (Figure 2), encompassing 87,891 bp in the large-copy region and 19,888 bp in the small single-copy region, with GC contents of 36.20% and 32.60%, respectively. The two inverted repeat regions are 22,150 bp in length with a GC content of 43.30%. A total of 126 genes have been identified, comprising 81 protein-coding genes, 37 tRNA genes, eight rRNA genes, and one pseudogene. Among these genes, four CDSs (*ndhB*, *rps7*, *rps12*, and *ycf2*), seven tRNAs (*trnA^UGC^*, *trnI^CAU^*, *trnI^GAU^*, *trnL^CAA^*, *trnN^GUU^*, *trnR^ACG^*, and *trnV^GAC^*), and four rRNAs (*rrn16*, *rrn23*, *rrn4.5* and *rrn5*) are duplicated in the IR regions. Additionally, 14 genes (*atpF*, *ndhA*, *ndhB*, *petB*, *petD*, *rpl16*, *rps16*, *rpoC1*, *ycf1*, *trnA^UGC^*, *trnG^UCC^*, *trnI^GAU^*, *trnK^UUU^*, *trnL^UAA^*, and *trnV^UAC^*) contain one intron each, while two genes (*clpP* and *ycf3*) contain two introns (Figure S2). The gene *rps12* consists of three exons, two of which are duplicated in the IRs (Figure S3).

**Figure 3.**
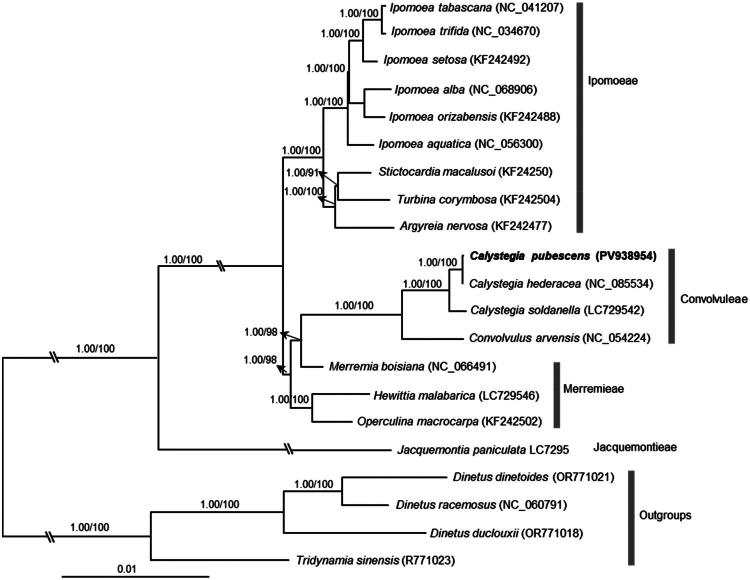
The Bayesian inference (BI) tree of 21 species inferred from the complete chloroplast genomes. Numbers on branches are the supporting values of BI and maximum parsimony, respectively. The *C. pubescens* ‘Anestia’ was marked in bold. Tribes of Convolvulaceae classification follow Stefanović et al. (2003). The following sequences were used: *Argyreia nervosa* KF242477 (Eserman et al. 2014), *Calystegia hederacea* NC_085534 (Fu et al. 2024), *Calystegia pubescens* PV938952 (this study)*, Calystegia soldanella* LC729542 (Wu et al. 2022), *Convolvulus arvensis* NC_054224 (Wang et al. 2021), *Dinetus dinetoides* OR771021 (Chen et al. 2024), *Dinetus duclouxii* OR771018 (Chen et al. 2024), *Dinetus racemosus* NC_060791 (https://www.ncbi.nlm.nih.gov/nuccore/NC_060791), *Hewittia malabarica* LC729546 (Wu et al. 2022), *Ipomoea alba* NC_068906 (Sudmoon et al. 2024), *Ipomoea aquatica* NC_056300 (Wang et al. 2021), *Ipomoea orizabensis* KF242488 (Eserman et al. 2014), *Ipomoea setosa* KF242492 (Eserman et al. 2014), *Ipomoea tabascana* NC_041207 (Sun et al. 2019), *Ipomoea trifida* NC_034670 (Zhou et al. 2018), *Jacquemontia paniculata* LC729558 (Wu et al. 2022), *Merremia boisiana* NC_066491 (https://www.ncbi.nlm.nih.gov/nuccore/NC_066491), *Operculina macrocarpa* KF242502 (Eserman et al. 2014), *Stictocardia macalusoi* KF242503 (Eserman et al. 2014), *Tridynamia sinensis* OR771023 (Chen et al. 2024), and *Turbina corymbosa* KF242504 (Eserman et al. 2014). The species in this study are highlighted in bold. The bar represented the nucleotide substitutional rate.

The BI and MP trees constructed from complete chloroplast genomes exhibited congruence (Figure 3). Both analyses indicated the monophyly of the three sampled *Calystegia* species, with *C. pubescens* ‘Anestia’ showing a close relationship to *C. hederacea*, supported by high values (Figure 3).

## Discussion and conclusions

Chloroplast genomes are essential for species identification, genetic diversity evaluation, and evolutionary research (e.g. Fu et al. 2024; Zhou et al. 2024; Qin et al. 2025). Typically, angiosperm chloroplast genomes range from 107 to 218 kb in length, containing 110–130 genes, with an average GC content of 30–45% (Wicke et al. 2011; Zhu et al. 2017). In this investigation, we conducted a novel assembly and annotation of the chloroplast genome of *C. pubescens* ‘Anestia’. The genome exhibited a typical quadripartite structure, spanning 152,079 bp, containing 126 identified genes, and possessing a GC content of 37.37%. The length, GC content, and gene composition of this chloroplast genome closely resembled those of *C. hederacea* (Fu et al. 2024) and *C. soldanella* (Wu et al. 2022), indicating a conservation of chloroplast genome characteristics within the *Calystegia* genus.

The phylogenetic analysis in this study confirms the monophyletic status of the genus *Calystegia* (Figure 3), consistent with previous studies (Stefanović et al. 2003; Chen et al. 2022; Wu et al. 2022). Our phylogenetic reconstruction reveals a close relationship between *C. pubescens* ‘Anestia’ and *C. hederacea*, supported by significant statistical values (Figure 3). In conclusion, our research contributes essential genetic information for further phylogenetic and evolutionary inquiries within the *Calystegia* genus.

## Supplementary Material

Supporting materials.docx

## Data Availability

The genome sequence data that support the findings of this study are openly available in GenBank of NCBI (https://www.ncbi.nlm.nih.gov/) under accession no. PV938954. The associated BioProject, SRA and Bio-Sample numbers are PRJNA1312251, SRR35187365, and SAMN50858762, respectively.
