# Supplementary material for: The complete chloroplast genome of Calystegia pubescens ‘Anestia’ Hara 1957 (Convolvulaceae), an endemic species in Asia
Source: Mitochondrial DNA B Resour. 2026 Jan 12;11(2):259–63. doi: 10.1080/23802359.2025.2602225 (PMC12798665; doi:10.1080/23802359.2025.2602225)

**Supporting material**

**Figure legends**

**Figure S1**. The mean and minimum read mapping depth of assembled chloroplast genome of *Calystegia pubescens* 'Anestia'

**Figure S2** The location of twelve genes with one intron or two introns.

**Figure S3** The location of *rps12* with three introns.

**Figure S1**. The coverage depth of the assembled chloroplast genome of *Calystegia pubescens* 'Anestia'. The horizontal coordinate was the positional information of the nucleotide acids and the vertical coordinate was the corresponding coverage depth of each base.


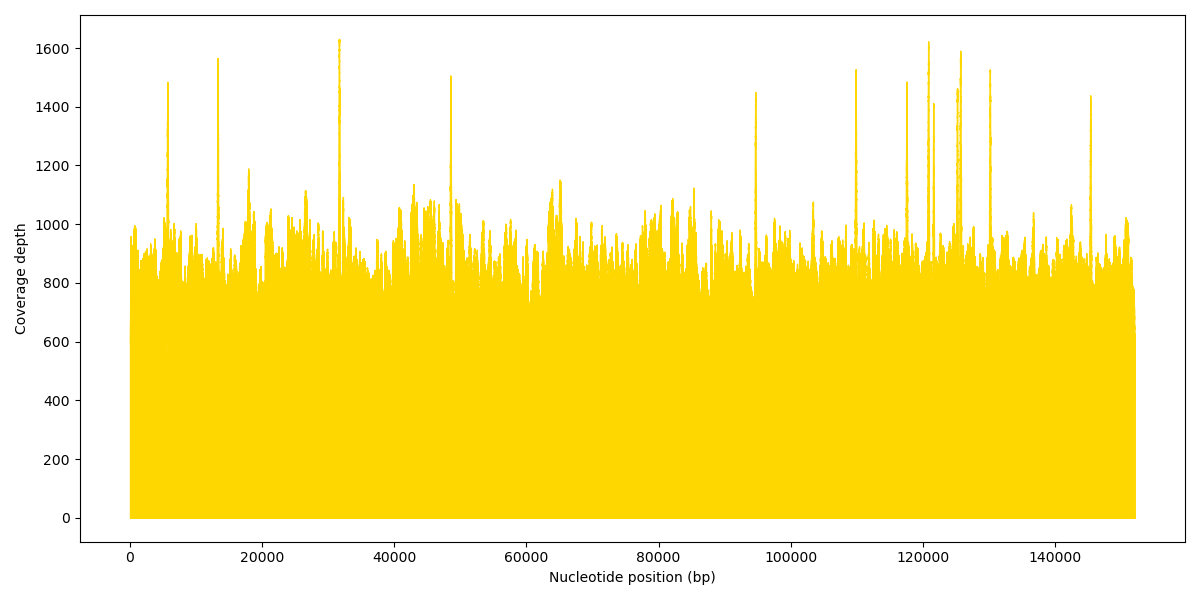


**Figure S2** Schematic diagram of cis-splicing genes in the plastome of *Calystegia pubescens* 'Anestia'. Among them, *rps16, atpF, rpoC1, petB, petD, rpl16, ndhB, ndhA* and *ycf1* contain on intron, while *ycf3* and *clpP* include two introns.


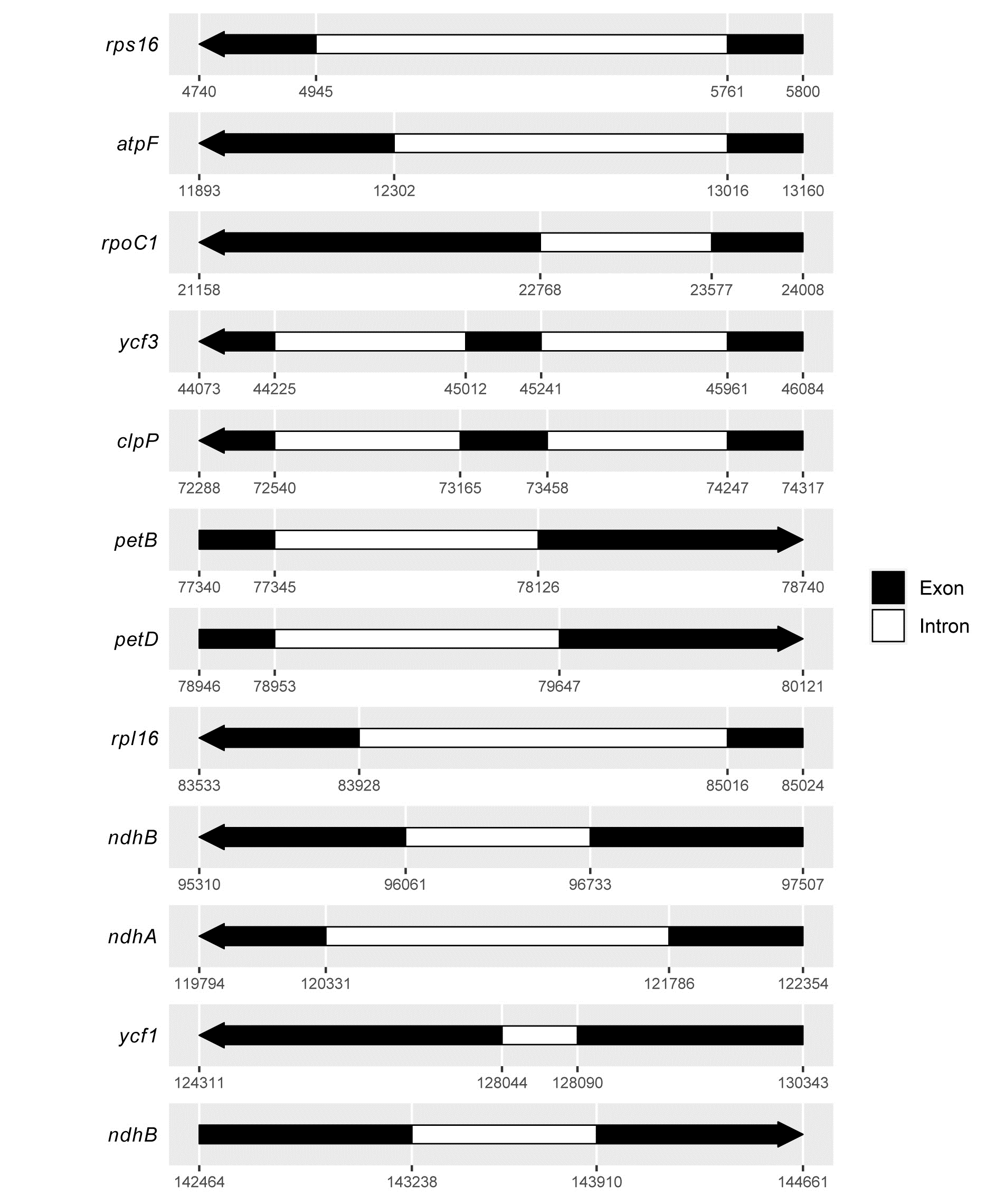


**Figure S3** Schematic diagram of trans-splicing genes *rps12* with three introns in the plastome of of *Calystegia pubescens* 'Anestia'.


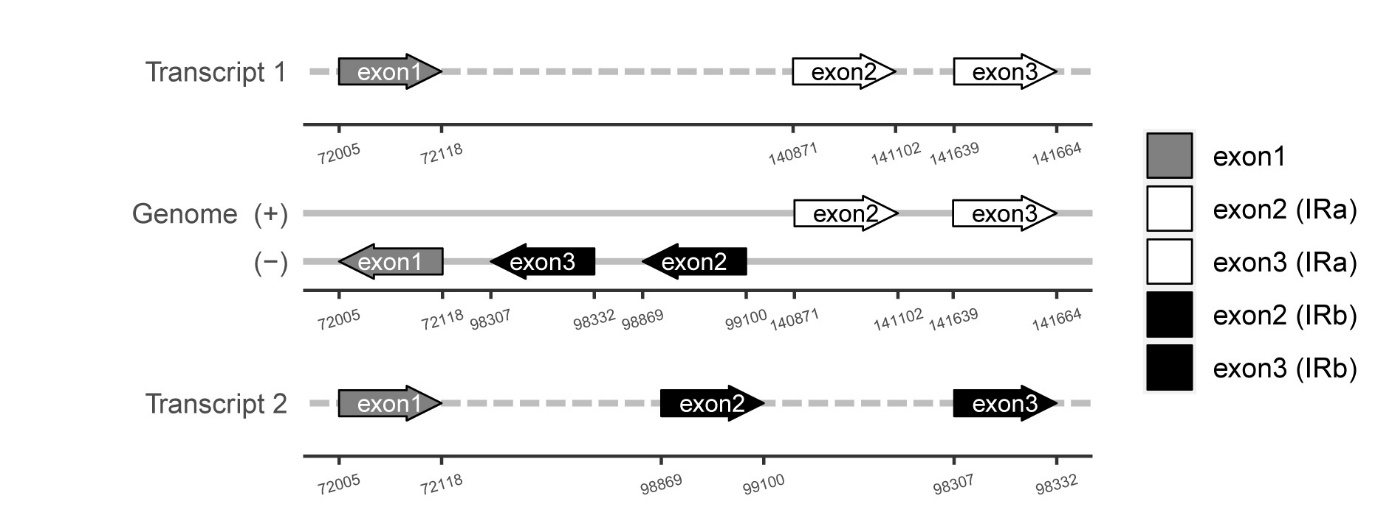

Supplement: Supporting materials.docx [file TMDN_A_2602225_SM8081.docx]
